# RGD-Labeled Hemocytes With High Migration Activity Display a Potential Immunomodulatory Role in the Pacific Oyster *Crassostrea gigas*

**DOI:** 10.3389/fimmu.2022.914899

**Published:** 2022-07-05

**Authors:** Zhao Lv, Limei Qiu, Weilin Wang, Zhaoqun Liu, Qing Liu, Lingling Wang, Linsheng Song

**Affiliations:** ^1^ CAS and Shandong Province Key Laboratory of Experimental Marine Biology, Institute of Oceanology, CAS Center for Ocean Mega-Science, Chinese Academy of Sciences, Qingdao, China; ^2^ Laboratory for Marine Biology and Biotechnology, Pilot National Laboratory for Marine Science and Technology (Qingdao), Qingdao, China; ^3^ Liaoning Key Laboratory of Marine Animal Immunology, Dalian Ocean University, Dalian, China; ^4^ Liaoning Key Laboratory of Marine Animal Immunology and Disease Control, Dalian Ocean University, Dalian, China

**Keywords:** *C. gigas*, RGD labeled hemocytes, migration activity, immunomodulatory, antimicrobial immunity

## Abstract

Immunocyte migration to infection sites is important for host cellular defense, but the main types of migrating hemocytes and their mechanisms against pathogen invasions are unclear in invertebrates. In the present study, a population of hemocytes in the Pacific oyster *Crassostrea gigas* labeled with a fluorescein isothiocyanate (FITC)-conjugated Arg-Gly-Asp (RGD)-containing peptide was sorted. RGD^+^ hemocytes were characterized by a smaller cell size and cytoplasmic-nucleo ratio, fewer cytoplasmic granules, and higher levels of myeloperoxidase, reactive oxygen species, and intracellular free calcium concentration. RGD^+^ hemocytes exhibited a high level of migration activity, which was further induced after *V. splendidus* infection. Transcriptome analysis revealed that RGD^+^ hemocytes highly expressed a series of migration-related genes, which together with migration-promoting genes were significantly upregulated after *V. splendidus* infection. The neuroendocrine system was also proven to regulate the migration activity of RGD^+^ hemocytes, especially with the excitatory neuroendocrine factor dopamine, which promoted migration activity as confirmed by receptor blocking assays. Meanwhile, RGD^+^ hemocytes could highly express immunomodulatory factor interleukin (IL)-17s and their receptor genes, which was positively related to the production of antimicrobial peptides in whole hemocytes after *V. splendidus* infection. Collectively, this study identified a specific hemocyte population, i.e., RGD^+^ hemocytes, that shows high migration activity in response to pathogen infection and exerts a potential immunomodulatory role by highly expressing IL-17s that might enhance the hemocytes’ antimicrobial peptide production in oysters.

## 1 Introduction

A unique property of the immune system that distinguishes it from other tissue systems in organisms is the migration of its major cellular components through body fluids into tissues and often back into the body fluids again to eliminate infectious pathogens, clear dead tissues, and repair the damage in hosts (1, 2). In mammals, neutrophils and macrophages are essential players of the innate immune system and are the first two types of leukocytes to migrate to the sites of infection to directly internalize and kill invading microbes or regulate immune responses by producing various cytokines, such as tumor necrosis factors (TNFs) and interleukins (ILs), that promote inflammation and antimicrobial effects (3–5). Several types of T lymphocytes, including CD8^+^ cytotoxic T cells, CD4^+^ helper T cells, and memory T-lymphocytes, serve as the key part of the adaptive immune system in mammals and have also been shown to migrate to infection sites and exert immune killing and regulation (6–8). Therefore, the migration of immunocytes is essential for both innate and adaptive immune responses and is considered an indispensable context of cellular immunity.

The molecular mechanisms of migration shared by different types of immunocytes have been extensively studied in mammals, among which a set of internal machineries mediated by integrins and integrin signaling pathways is reported as an essential requirement (9–11). It is generally believed that rapid actin polymerization drives protrusions at the front edge of migrating immunocytes, and the activation of molecular motor myosin provides contractile forces that lead to contractions at the rear of migrating immunocytes, ultimately forcing the cells to move forward (12). During this process, the linkage of integrins’ cytoplasmic region to intracellular actins is directly associated with the activity of actin polymerization and integrins on the cell surface that interact with extracellular matrix molecule (ECM) ligands also increase cell adhesions and finally promote protrusions at the front edge of migrating immunocytes (13, 14). The activation of integrin signaling pathway molecules such as PI3K, phosphatidylinositol-3-phosphate (PIP3), and the small GTPases Rac and Cdc42 can enhance the activity of actin polymerization for the protrusions at the front edge of migrating immunocytes (15, 16). Furthermore, the molecules that regulate integrin signaling pathways, such as the small GTPase Rho at the rear of migrating immunocytes, have been demonstrated to activate ROCKs and MLCKs to ultimately promote myosin motor activity (17, 18), indicating that integrins and integrin signaling pathways also play important roles in the contractions at the rear and subsequent detachment of migrating immunocytes.

Invertebrates lack the T and B cells necessary for adaptive immunity, and circulating hemocytes are thought to be the most crucial cellular component of innate immunity for the recognition and removal of foreign invaders (19–21). An increasing body of evidence has suggested that the migration of hemocytes plays important roles in innate immune defenses in many invertebrate species. In the fruit fly *Drosophila melanogaster*, for example, hemocytes have been observed to migrate at wound sites and engulf cell debris under scanning electron microscopy (22, 23). The hemocytes of shrimp *Litopenaeus stylirostris* have also been shown to migrate toward the infection sites following the challenge of *Vibrio penaeicida*, with a local and massive release of penaeidin that exerts antimicrobial effects (24). In addition, a number of integrin signaling pathway genes, such as the small GTPases Rho and Rac3 and cytoskeleton dynein, are significantly upregulated in the Manila clam *Ruditapes philippinarum* following challenge with *V. alginolyticus* based on an immune-enriched oligo-microarray analysis (25), suggesting a conserved molecular basis for hemocyte migration in invertebrates.

Nevertheless, the main types of hemocytes that migrate at the sites of infection to exert immune functions are still unknown, as studies on cell typing are greatly impeded due to the lack of molecular markers and effective monoclonal antibodies in invertebrates (26–28). In mammals, the tripeptide RGD is a specific ligand for RGD-binding integrins, including α5β1, α8β1, αVβ1, αVβ3, αVβ5, αVβ6, αVβ8, and αIIbβ3 (29). Given the key roles of these integrins in cell migration, fluorescent RGD-containing peptide (RGDCP) has been widely employed as the specific probe for integrins to image certain migrating cells. For example, RGDCP conjugated with the fluorescent dye FITC, Cy7, or Cy5 is available for targeting integrin αVβ3, which is highly expressed on the glioma cell surface, to indicate the migration activity, morphological change, and invasion processes (30–32). Considering that synthetic RGDCP has also been reported to conservatively bind hemocytes *via* surface-specific integrins in many invertebrates, including *D. melanogaster* (33), *Botryllus schlosseri* (34), *Pacifastacus leniusculus* (35), *Lymnaea stagnalis* (36), *Mytilus trossulus* (37), and *C. gigas* (38–40), fluorescent RGDCP as a specific probe for invertebrate integrins may also be practical for marking a population of hemocytes and helpful for studying the main types of migrating hemocytes and their roles in the immune responses of invertebrates.

The Pacific oyster, *C. gigas*, is one of the most important mariculture bivalve species worldwide (41). As a sessile filter feeder exposed to a wide range of biotic and abiotic stresses, *C. gigas* represents an attractive model for studying the immunologic mechanisms for stress adaptation in invertebrates (41). Molecular immunology research has made great strides after the identification of most immune genes, but there are still many unknowns in the cellular immunity of this animal (42–44). The involvement of integrins in cellular immune responses and the binding of RGDCP to integrins and hemocytes in *C. gigas* were reported in our previous studies (38, 39). In this study, RGDCP-bound positive hemocytes (RGD^+^ hemocytes) were sorted by fluorescence-activated cell sorting (FACS), and the labeled cells were proven to have high migration activity. Therefore, the objectives of the study are to (1) analyze the features of cellular morphology and molecular characteristics of RGD^+^ hemocytes, (2) investigate the molecular mechanisms underlying the high migration activity of RGD^+^ hemocytes in response to the pathogen *V. splendidus*, and (3) explore the possible roles of RGD^+^ hemocytes after their migration at the infection sites in *C. gigas*. The study will offer new insights into the main types of migrating hemocytes and their immune defense mechanisms against microbial infection in *C. gigas*.

## 2 Materials and Methods

### 2.1 Animal Rearing and Manipulation

Pacific oyster (*C. gigas*) specimens with lengths of 10~15 cm and weights of 150~200 g were collected from a local breeding farm in Rongcheng, China. These specimens were acclimated in aerated and filtered seawater (with a salinity of approximately 32.0‰) at 18°C and fed spirulina powder every other day for 2 weeks prior to use in experiments. For immune challenge, the oysters were stimulated with 100 μl of *V. splendidus* (10^6^ cells/ml, strain JZ6 provided by Rui Liu from the Institute of Oceanology, Chinese Academy of Sciences) by injection into adductor muscle for 12 or 24 h followed by hemocyte extraction. Seawater in the aquaria was replaced every day, and all experiments involving animals reported in this study were approved by the Ethics Committee of the Institute of Oceanology, Chinese Academy of Sciences.

### 2.2 Hemocyte Preparation

Approximately 1 ml of hemolymph per oyster was extracted from the adult *C. gigas* specimens by using a sterile syringe needle (with a diameter of 0.7 mm) to carefully puncture the pericardial cavity and to avoid poking the gonads and other tissues to ensure that the obtained hemolymph was free from contamination by other type cells. The hemolymph was immediately mixed with prechilled anticoagulant acid citrate dextrose solution (7.3 g/l citric acid, 22.0 g/l sodium citrate, and 24.5 g/l dextrose) at a 7:1 volume/volume ratio, pooled into sterilized 50-ml Falcon tubes, pelleted at 800 × *g* at 4°C for 5 min, and washed twice with modified Leibovitz L15 medium (supplemented with 0.54 g/l KCl, 0.6 g/l CaCl_2_, 1 g/l MgSO_4_, 3.9 g/l MgCl_2_, 20.2 g/l NaCl, 100 U/ml penicillin G, 40 µg/l gentamycin, 100 µg/ml streptomycin, 0.1 µg/ml amphotericin B, and 10% fetal bovine serum) (45). Hemocytes were resuspended in modified Leibovitz L15 medium after using a screen mesh with a pore size of 70 μm to remove the larger non-cellular particles and stored on ice for 30 min to reduce spontaneous aggregation.

### 2.3 Labeling and FACS of RGD^+^ Hemocytes

The FITC-conjugated RGDCP peptide was synthesized by Sangon Biotech (Shanghai, China) and used as the probe for certain integrins to label specific populations of oyster hemocytes as previously described (38, 39). Briefly, the collected hemocytes were resuspended in modified Leibovitz L15 medium and incubated with FITC conjugated-RGDCP (0.01 mg/ml) for 30 min. After three washes with modified Leibovitz L15 medium, some hemocytes with FITC conjugated-RGDCP were fixed on glass slides, dyed with DAPI, and observed under a laser confocal scanning microscope (Carl Zeiss LSM 710).

**Figure 1:**
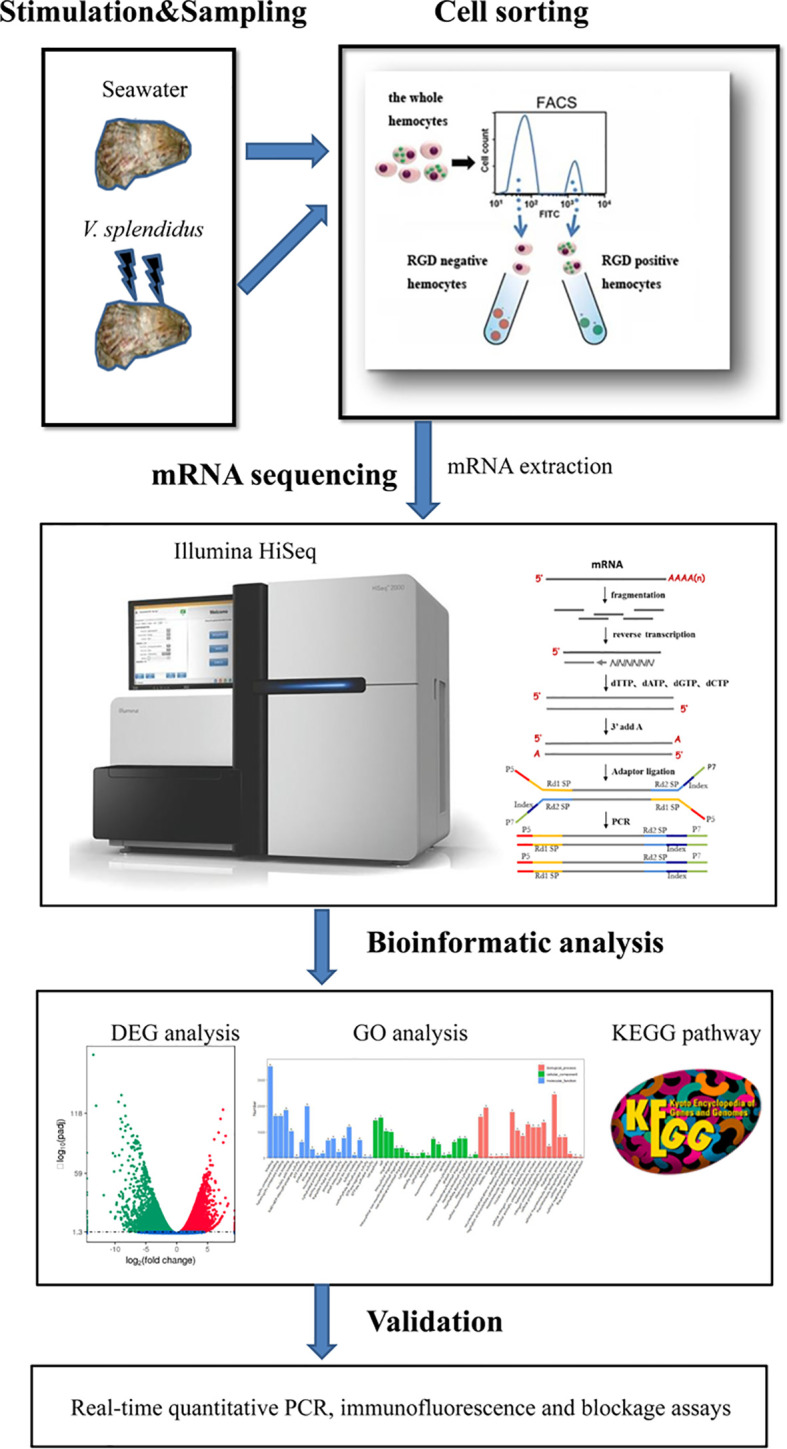
Pipeline overview of analyses of the molecular mechanisms for the mediation of high migration activity and regulation of antimicrobial immunity of *C. gigas* RGD^+^ hemocytes. The hemocytes were collected, incubated with FITC-conjugated RGDCP, and sorted using FACS to prepare both RGD^+^ hemocytes and RGD^-^ hemocytes. The differential expression in RGD^+^ hemocytes before or after *V. spelendidus infection* was revealed by transcriptome bioinformatic analyses, including DEG, GO term, and KEGG pathway analyses. The molecular mechanisms for the mediation of high migration activity and regulation of antimicrobial immunity were revealed based on DEGs and further validated using qPCR, receptor blocking assay, and confocal microscopy.

The percentage of hemocytes with FITC-conjugated RGDCP was analyzed by flow cytometry (BD FACSAria II). FITC-positive and -negative cells were gated using an FL1 channel, and the population of hemocytes with green signals derived from FITC-conjugated RGDCP was designated RGD-bound positive hemocytes (RGD^+^ hemocytes), while the other mixture of hemocyte populations was assigned RGD-bound negative hemocytes (RGD^-^ hemocytes). To explore the characteristics of oyster RGD^+^ hemocytes and their responses to *V. splendidus* infection, resting RGD^+^ and RGD^-^ hemocytes (from blank oysters without any stimulations) and activated RGD^+^ and RGD^-^ hemocytes (from oysters stimulated by *V. splendidus* after 24 h) were sorted using FACS based on the fluorescence intensity, followed by cytochemical staining, cellular function, and transcriptomic analyses (**Figure 1**).

### 2.4 Cytochemical Staining Analysis

The resting RGD^+^ and RGD^-^ hemocytes were collected and plated onto glass slides to allow cell adhesion at 18°C for 3 h. Then, hemocytes were fixed with 4% PFA and stained with Wright, Giemsa, and hematoxylin–eosin (HE) as previously described (46). For myeloperoxidase (MPO) staining, hemocytes were fixed with 10% formal ethanol and stained with MPO for 10 min. The morphological and cytochemical characteristics of hemocytes were observed by light microscopy (Olympus, Tokyo, Japan).

### 2.5 Detection of Intracellular ROS and Ca^2+^ Levels

The resting RGD^+^ and RGD^-^ hemocytes were washed twice with modified Leibovitz L15 medium, and the concentration was adjusted to 1 × 10^6^ cells/ml. The specific fluorescent probe DHE or Rhod-2 AM (Beyotime, Shanghai, China) was diluted to 1 μM, mixed with the resting RGD^+^ and RGD^-^ hemocyte suspensions, respectively, and incubated for 45 min at room temperature in a rotary mixer according to the instructions for the detection of intracellular ROS or Ca^2+^. The stained resting RGD^+^ and RGD^-^ hemocytes were collected at 800 g and 4°C for 10 min by centrifugation and washed three times with modified Leibovitz L15 medium. The fluorescence signal of the probes was detected by flow cytometry, and the mean fluorescence intensity (MFI) value was used to reflect the intracellular ROS (or Ca^2+^) levels in different populations of hemocytes. There were three replicates for each sample.

### 2.6 Library Preparation and RNA Sequencing

To explore the molecular mechanisms of oyster RGD^+^ hemocytes and their responses against *V. splendidus*, nine RNA-seq paired-end libraries from the sorted resting RGD^+^, resting RGD^-^, and activated RGD^+^ hemocytes were generated using the NEBNext^®^ Ultra^™^ RNA LibraryPrep Kit for Illumina^®^ (NEB, Ipswich, MA, USA) following the manufacturer’s procedure (47). Sequencing of libraries was performed using an Illumina HiSeq 2000 platform, and 150-bp paired-end raw reads were generated. After removing adapter sequences, poly-N sequences, and low-quality reads, 7.12~8.41-G clean reads were obtained in each library (**Table S1**). The average Q20 of sequencing and the alignment ratio of sequenced nucleotides against the reference genome (http://www.oysterdb.com) were greater than 96% and 71%, respectively (**Table S2**). Three biological transcriptomic sequencing replicates were performed.

### 2.7 Transcriptome Bioinformatic Analysis

Differential expression analyses were performed between the resting RGD^+^ and RGD^-^ hemocytes and between the activated and resting RGD^+^ hemocytes using the DEGseq R package (1.10.1) (48). Benjamini and Hochberg’s approach (49) was used to control the false discovery rate (FDR) by resulting P value adjustment. Genes with an adjusted P value (or q value) <0.05 found by DEGseq were selected as differentially expressed. Gene Ontology (GO) enrichment was then analyzed using DAVID 6.8 Beta (https://david.ncifcrf.gov), and the results were classified with GOTERM_BP, GOTERM_CC, and GOTERM_MF (50). Significantly enriched signal transduction pathways represented by differentially expressed genes (DEGs) were determined using KEGG pathway enrichment analysis compared with the whole-genome background (51). All sequences obtained in this study are deposited in GenBank (PRJNA826541), and the analyzed data of DEGs are listed in **Tables S3** **–** **4**.

### 2.8 qPCR for Gene Expression Analysis

The resting RGD^+^, resting RGD^-^ hemocyte, activated RGD^+^, and activated RGD^-^ hemocyte cDNAs were prepared as described previously (38). For validation of transcriptomic analyses, representative DEGs involved in hemocyte migration, neuroendocrine regulation, and immunomodulation were selected from transcriptomic data, and their mRNA expression profiles were determined by using SYBR Green fluorescent qPCR. In addition, six reported antimicrobial peptides (AMPs), including *Cg*-BigDefensin-1~3, *Cg*-Defensin-1~2, and *Cg*-BPI, were also detected according to references (52, 53) to explore the potential antimicrobial roles of the different hemocyte populations in oysters. SYBR Green fluorescent qPCR was performed using an ABI PRISM 7500 Sequence Detection System (Applied Biosystems, Foster City, CA, USA) following the manufacturer’s protocols. The relative gene expression levels were analyzed by the 2^−ΔΔCT^ method (54) with the oyster encoding elongation factor 1 (*Cg*EF-1) as the internal control (55). All data are presented in terms of relative gene expression (N = 6). Detailed information on the primer nucleotide sequences is summarized in **Table 1**. The dissociation curve analysis of amplification products with technical repetitions was performed at the end of each PCR to confirm that only one PCR product was amplified and detected, which reflected the specificity and efficiency of the primer pairs.

**Table 1:** The information of representative DEGs associated with hemocytes migration, neuroendocrine regulation and immunomodulatory, and AMP genes for qPCR validation.

Functional descriptions	Molecular categories	Accession numbers	Forward primer sequences	Reverse primer sequences
**Myosin activating molecules**	ROCK	CGI_10004418^*^	GAAGTGGGCTAAGTTCTAC	AATTTCAATGTCTGTGGG
	MLCK	CGI_10016803^*^	ACAGTATCAATAAGCACGAC	AATGACAGATGATGTTTCG
		CGI_10015855^**^	GATGATGGAGTCCGCTAA	TGAGGTGGTGTTTCGTAT
		CGI_10016804^**^	TCGGAGGTGATTAGGACG	GGCAGTATGCTGGAGGGT
**Activation of molecules at edge**	Rho	CGI_10019637^*^	ATGGCAACTTCGGTACTTC	GGATTCAGCACCCTTTCA
		CGI_10028030^&^	GATTCATTACGCTTGATAAG	TGAGGTTCATTTAGTGCC
		CGI_10012684^&^	AGGAGATGGTGCTTGTGG	TTGCGATTTGACTGGTTC
**Actin polymerization**	Actin	CGI_10020997^&^	AGAGCCTGGCAGATAAGA	TTGTTGAGATAGACGGTTG
		CGI_10000602^&^	CCACTCAATCCCAAATACA	GCCTGCTAGGTCCACTCT
		CGI_10024579^&^	ATGGGACAGAAGGACAGC	GGCGTAACCCTCGTAGAT
		CGI_10003492^&^	GATTTGACCGATTACCTTAT	TCCAGACCGAGTATTTCC
**Activation of molecules at front**	PI3K	CGI_10027824^&^	ATGCCTCCGATAGTGGTC	TCTCATCCTTCTGTCCCT
	GTPase	CGI_10000237^&^	TGTGACGGTGATGATTGG	GGCGATACTACGGAGAAG
		CGI_10021345^&^	AATGCTCTGCCCTTACCC	CACAGCCTTTCTTCGTTT
		CGI_10021938^&^	GGCAGGAGGATTACGACA	TTTCACAGGCGACAGACC
	Integrin	CGI_10012356^*^	TGGGAAGTTGGCTAAGGA	ACAGATGACAAAGCGTGA
		CGI_10013155^*^	TGTATCTGATGCAGACCGAGTC	GGTGGTGCTGGTTGGAAT
		CGI_10008246^*^	AGTGGCGGAATCTGTGAC	GGTGATGTTTGCCTGTATGA
		CGI_10017565^*^	CCCTTGGGTACTACCTTT	TAATCCCTTTCCAGACAC
		CGI_10021392^&^	TGTTTACTGGGTGGTTTG	ATCTCCTGTGGCTCCTAC
		CGI_10021391^&^	CATCCACGGGAGGAACTT	CGAAACCGCCACCTATCT
		CGI_10021257^*^	TGGCTTTAATGTCCCACC	CATCAACAGGCAAATACAAA
		CGI_10012568^&^	GTGAGGCACTCTTAACCA	AGGGAAATAAATGTAGCG
		CGI_10023513^&^	GTGGAGCCTGGGAGTTAC	TTTGGACCCTTCTAATGT
		CGI_10009281^&^	TGAGCACCATCACTAAGAA	CATTGACTTTGTCCCTGA
		CGI_10014761^&^	TTATTGGTGGTGATGAGTC	GGGCAGGTATTTAGGTTA
		CGI_10012179^&^	ACCCATCAGTTGGTCAGA	CTCGTAGACGCCTGTAGC
		CGI_10012180^&^	TACTCCTATCGCCATCGT	TTTCCCTCCATCCAATCT
		CGI_10009280^&^	CGCAGAAGGGCACTAATA	ACAGACTCCGCAGATACA
		CGI_10012178^&^	GTAACGCCAACTACACGG	ACACTGACAGCGACCACA
**ECM-integrin interation**	ECM	CGI_10025801^&^	GGGCGAACAAGGACGAAC	CCAGGCAAGCCAAGCAAT
		CGI_10013230^&^	GGCTTCCATCACTTACAC	GATTACTTACTACAGCGACA
		CGI_10003195^&^	CCGACCAAGTTTACAATG	GTAGTAGGTGGAGGCAGT
		CGI_10018037^&^	AGGCATTAGAGTCACCCG	GCCCACCTGAGTATTGTAG
		CGI_10002428^**^	TGGTTCCGACTGTGCTAA	GGTGTAACGTCCATCCTC
		CGI_10006107^**^	TTCAGGCACAGACCGCTAA	GGCAGAGGAATCGACCAG
		CGI_10010799^*^	TAACAGAAACGCAGAAACC	TCACCATAATAACCAGCATC
		CGI_10019046^*^	CCACAGAAACCAGTGCCTAT	CCATCCGTGTTGTCCCTA
		CGI_10021302^*^	GCAACTGTCTGCCAGGAT	CCAGGCTTACACTTCTCAC
		CGI_10013234^&^	GGGTTTAACTGCAATCGA	CCACTGAAGGCTTGTTCT
		CGI_10001011^**^	CTTCAGCACCTCTTGTCA	TTTCAGACGCTGTTACCC
		CGI_10002890^**^	CGGACACTATGTTGGAAG	ACAAGAGGTGCTGAAGAA
		CGI_10023871^**^	ACGTCGGCGATGGGTATA	CGCACGCATTGAGATTTG
		CGI_10015959^**^	TGTTCTACCAAGACCCGTAT	TTAGTGACCGTCCCAATC
		CGI_10026821^**^	AGACCCGAGCCTCACAGA	TGGCAGCATTAAGAGCATT
		CGI_10010439^&^	CCGTGATGCTTGATTTCT	CAGGTGGTTGTTGATGCTTA
		CGI_10013675^*^	CCCTAACGGACAGTACACCG	GACGGATCATCATCTGCA
		CGI_10010465^*^	AAATACCAGGGAAGACCA	TAACCGTGCCTACAAACA
		CGI_10013991^*^	GTAAATCGGACAACTGCC	CAATTATCCTGCTCATCGT
		CGI_10012341^&^	ACAGCGTGATTGTTCTTCT	TGGTCAGGGTGTAGTTCG
		CGI_10022338^*^	AAGAGCTGCGAGTTGATA	TTAGGGTTGGCATAGAAA
**Neuroendocrine factor receptors**	DRD	CGI_10010352^&^	GAACGAAAGCAGCACCAC	GAAGGCGTAAGTCCGATG
		CGI_10014188^&^	TCATCACTTCCGTTAGCA	ACCAATACCATCACCACC
	ADR	CGI_10015982^&^	AGGGCATTGAATCCAGTC	TGTTTGCGGAGGAGAAAT
		CGI_10014189^&^	GCCGAACTCCTGCTCCTA	CACGCTTGGTAATACTGCTCTT
	GABR	CGI_10013184^&^	GGTCGTAGGGTCAAAGGG	AGTCCGAGTGCTGGTGCT
		CGI_10027781^&^	TGGCTGGCATTTCTGTTG	GAAGGTGGGAGCGGATAA
**Immunomodulatory factors**	IL-17	CGI_10025754^&^	CCCACGAATCTTGCTGAA	GGGACGCTACGAGGAAAT
	IL-17R	CGI_10021486^&^	AAAAGTCGCTGATAAAGG	CTGCTTGGTCCATAGAGT
		CGI_10000871^&^	GGACGACTGGTTGATTGA	CACAGCGACAGCAAAGTA
		CGI_10002512^&^	GTCTCGGTTCATCGTAGC	GATTGCCAAACTTGACACTA
**Antimicrobial peptides**	Cg-BPI	AY165040	AGTGCACAGTCGAAGGAAGG	AAGCCGGGGGTCTTACATTG
	Cg-BigDefensin-1	JF703138.1	TTTTGGTTTCGCCTGCTTCC	CAGCCCTGCGTAAGATGCTA
	Cg-BigDefensin-2	JF703145.1	GTGCAGACCGACCTGCTATT	GGTCTGCAGCACCTGTATGA
	Cg-BigDefensin-3	JF703148.1	TGACAGTCATTCGTGTGCCA	CCAAACGTGTTTGCCCAGTC
	Cg-Defensin-1	FJ669422.1	TCCGGGTGACCAGTATGAGT	ACAGCATTCATTGCTATCCCA
	Cg-Defensin-2	FJ669345.1	TTGGTCGTTCTCCTGATGGT	CAGTAGCCCGCTCTACAACC
**Reference gene**	CgEF-1	AB122066	AGTCACCAAGGCTGCACAGAAAG	TCCGACGTATTTCTTTGCGATGT

The genes with the superscripts of “*”, “**”, and “&” stood for the DEGs selected for validating the differential expressions in the resting RGD^+^ hemocytes and RGD^-^ hemocytes (“*”), in the activated RGD^+^ hemocytes and resting RGD^+^ hemocytes (“**”), and both in resting RGD^+^ hemocytes and RGD^-^ hemocytes and in the activated RGD^+^ hemocytes and resting RGD^+^ hemocytes (“&”), respectively. Their primer nucleotide sequences were shown.

### 2.9 The Detection of Migration Rate

Cell migration rates of the resting RGD^+^, resting RGD^-^, activated RGD^+^, and activated RGD^-^ hemocytes were measured by using EMD Millipore MultiScreen™ 96-well assay plates (Millipore, pore size: 5.0 μm) based on a previous description (56). Briefly, the prepared hemocytes were first washed twice with modified Leibovitz L15 medium and resuspended at 5 × 10^6^ cells/ml. Then, calcein AM (Invitrogen, Carlsbad, CA, USA) was added to the suspension at a final concentration of 5 μM to label the cells. After incubation at room temperature for 30 min, the cells were resuspended in 50 μl modified Leibovitz L15 medium and planted in the inserts of the plate whose receiver well was previously added to 150 μl modified Leibovitz L15 medium with 10% fetal bovine serum (FBS, TransGen, Peking, China). The fluorescence of each well was then measured to indicate the total number of hemocytes (Ex/Em = 494/517). After 60 min of incubation, the undersides of the inserts and the receiver were carefully washed and swabbed using modified Leibovitz L15 medium to remove all migrated cells. The fluorescence of each well was measured again to indicate the total number of non-migrants. Migration rates were subsequently calculated after all trials were conducted with three replicates. Cells seeded into plates with modified Leibovitz L15 medium and without FBS were used as negative controls. For the dopamine receptor blocking assay, the resting RGD^+^ and activated RGD^+^ hemocytes were previously incubated with SCH 23390 (specific antagonist for D1DR) for 1 h, followed by the detection of migration rates. There were three replicates for each sample.

### 2.10 Immunofluorescence Analysis

The expression changes of representative IL-17 (*Cg*IL17-1) (57) at the protein level in RGD^+^ hemocytes after *V. splendidus* challenge were analyzed by immunofluorescence assay. In brief, resting RGD^+^ hemocytes and activated RGD^+^ hemocytes were plated onto glass-bottom culture dishes for 1 h of cell adhesion. Cells were washed with PBS and fixed with 4% PFA at room temperature for 15 min before they were permeabilized with 0.1% Triton X-100 for 10 min. Cells were blocked with 3% BSA in PBS-Tween for 1 h and incubated with the mouse antiserum against *Cg*IL17-1 (46, 58) previously prepared in our lab (1:100-diluted in PBS-Tween) at room temperature for 1 h. Then, hemocytes were extensively washed with PBS-Tween and incubated with Alexa Fluor 594-labeled goat anti-mouse IgG (1:500-diluted in PBS-Tween) for 1 h, followed by incubation in DAPI (US Everbright, Inc., Greenfield, WI, USA) for 10 min. The hemocytes were washed, and fluorescence images were captured using a Carl Zeiss LSM 710 confocal microscope (Carl Zeiss, Germany). The red fluorescence intensity of Alexa Fluor 594 was analyzed by Zeiss software to reflect the changes in *Cg*IL17-1 protein expression. The unimmunized mouse serum and commercial Alexa Fluor 594-labeled goat anti-mouse IgG were used as the controls for the primary and secondary antibodies, respectively.

### 2.11 Statistical Analysis

A two-sample Student’s t-test was used for all comparisons between groups in this study. Statistical analyses were carried out using GraphPad Prism 5 software, and all results were reported as the means ± SD. Statistical significance was defined at *p* < 0.05.

## 3 Results

### 3.1 The Labeling of RGD^+^ Hemocytes and Their Response to *V. splendidus* Challenge

**Figure 2:**
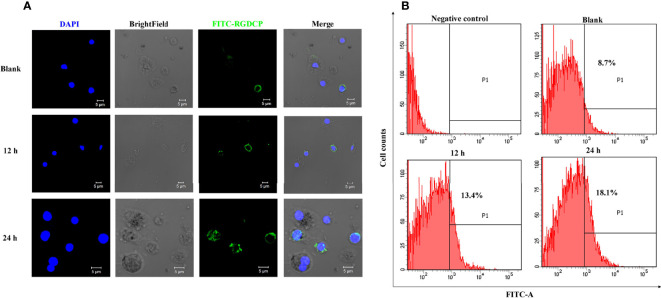
Fluorescent labeling of *C. gigas* RGD^+^ hemocytes and their response to *V. splendidus* challenge. **(A)** Immunofluorescence analysis of numerary changes in *C. gigas* RGD^+^ hemocytes after stimulation with *V. splendidus.* FITC-conjugated RGDCP-labeled oyster integrins on cell membranes are indicated in green, and the cell nucleus stained with DAPI is indicated in blue. Bar = 5 μm. **(B)** Flow cytometry analysis of numerary changes in *C. gigas* RGD^+^ hemocytes after stimulation with *V. splendidus.* FITC-positive cells and RGD^+^ hemocytes were gated as P1.

Confocal microscopic analysis revealed that FITC-conjugated RGDCP could label a small portion of hemocytes (RGD^+^ hemocytes) from the oyster *C. gigas* with a circle of green fluorescent signals on their surfaces (**Figure 2A**). Flow cytometric analysis showed that RGD^+^ hemocytes accounted for 8.7% of the whole hemocytes in the blank oysters (designated resting RGD^+^ hemocytes, **Figure 2B**), which was consistent with previously reported results (39). The hemocytes with negative signals accounted for the majority of oyster hemocytes representing multiple cell types and were assigned as RGD^-^ hemocytes (**Figure 2B**). After challenge with *V. splendidus* for 12 and 24 h, the percentage of RGD^+^ hemocytes significantly increased to 13.4% and 18.1%, respectively (**Figure 2B**).

To investigate the morphological and functional features of RGD^+^ hemocytes, resting RGD^+^ hemocytes and RGD^+^ hemocytes from oysters stimulated with *V. splendidus* for 24 h (designated activated RGD^+^ hemocytes) were obtained by using FACS. The sorting purities were 88.6% and 98.5% for the resting RGD^+^ hemocytes and the activated RGD^+^ hemocytes, respectively (**Figure S1**).

### 3.2 Morphological and Biochemical Features of RGD^+^ Hemocytes

**Figure 3:**
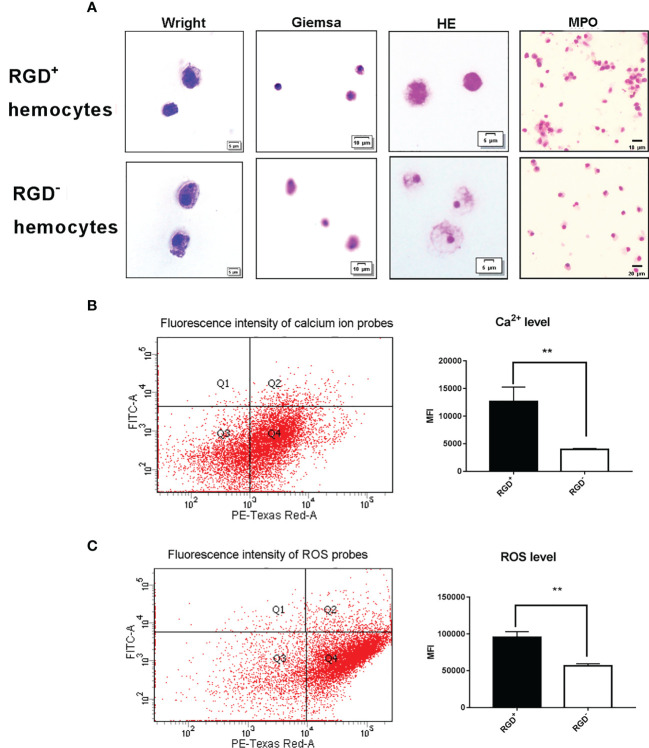
Morphological and cytochemical features of *C. gigas* RGD^+^ hemocytes. **(A)** The cytochemical staining of resting RGD^+^/RGD^-^ hemocytes. Resting RGD^+^/RGD^-^ hemocytes were stained for Wright, Giemsa, HE, and MPO. The upper panel and lower panel show cytochemical staining of sorted resting RGD^+^ hemocytes and RGD^-^ hemocytes, respectively. The differences in **(B)** intracellular Ca^2+^ and **(C)** ROS levels between resting RGD^+^ and RGD^-^ hemocytes. The double asterisk (**) stands for statistical significance at *p* < 0.01.

The cytochemical staining of the resting RGD^+^ hemocytes and RGD^-^ hemocytes was compared to reveal the morphological features of RGD^+^ hemocytes. Wright, Giemsa, and HE staining revealed that the resting RGD^+^ hemocytes represented the population of smaller cells 5–10 µm in diameter in the whole oyster hemocytes, while most resting RGD^-^ hemocytes were 8–15 µm in diameter (**Figure 3A**). Meanwhile, the resting RGD^+^ hemocytes were characterized by a relatively smaller cytoplasmic–nucleo (C: N) ratio and fewer large granules in the cytoplasm than the resting RGD^-^ hemocytes (**Figure 3A**). For MPO staining, the positive brown precipitates replenished the cytoplasm of the resting RGD^+^ hemocytes but regionally in the resting RGD^-^ hemocytes (**Figure 3A**).

Flow cytometric analysis was conducted to compare the differences in the levels of Ca^2+^ and ROS between resting RGD^+^ hemocytes and RGD^-^ hemocytes (**Figure 3B**). The Ca^2+^ level indicated by the MFI value was 1.27 × 10^4^ in the resting RGD^+^ hemocytes, and it was significantly higher than that of 0.40 × 10^4^ in the resting RGD^-^ hemocytes (*p* < 0.05). The ROS level indicated by the MFI value was 9.56 × 10^4^ in the resting RGD^+^ hemocytes, and it was also significantly higher than that of 5.67 × 10^4^ in the resting RGD^-^ hemocytes (*p* < 0.05, **Figure 3C**). These results indicated that RGD^+^ hemocytes were a type of cell with a smaller size and C:N ratio, fewer large granules, and higher levels of myeloperoxidase, Ca^2+^, and ROS in the cytoplasm.

### 3.3 The Migration Activity of RGD^+^ Hemocytes Before or After *V. splendidus* Challenge

**Figure 4:**
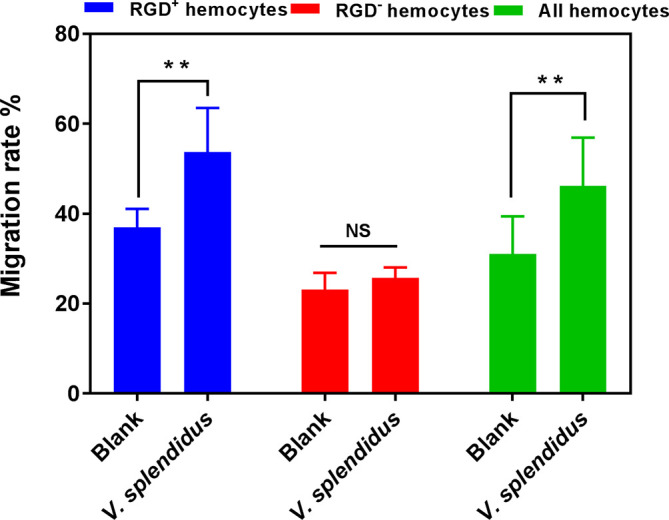
The migration activity of RGD^+^ hemocytes before and after *V. splendidus* stimulation. The double asterisk (**) stands for statistical significance at *p* < 0.01, and “NS” represents no statistical significance.

Given the key roles of RGD-binding integrins in cell migration, the migration activity of RGD^+^ hemocytes was detected to reveal their functional characteristics in *C. gigas*. The results showed that the migration rate of the resting RGD^+^ hemocytes was significantly higher than that of the resting RGD^-^ hemocytes before challenge with *V. splendidus* (36.98% vs. 23.12%, *p* < 0.01, **Figure 4**). After challenge with *V. splendidus* for 24 h, the migration rate of whole oyster hemocytes significantly increased from 31.04% to 46.25% compared to that of the blank group (*p* < 0.01, **Figure 4**). The migration rate of the activated RGD^+^ hemocytes (53.75%) was also significantly higher than that in the resting RGD^+^ hemocytes of 36.98% (*p* < 0.01, **Figure 4**), while the migration rate of RGD^-^ hemocytes was 25.74% after challenge with *V. splendidus* for 24 h, which was not significantly different from that in the blank group oysters of 23.12% (*p* > 0.05, **Figure 4**). These results indicated that the RGD^+^ hemocytes showed high migration activity before challenge with *V. splendidus*, and the migration activity could be further induced after challenge with *V. splendidus*.

### 3.4 Comparative Transcriptome Analysis of Resting RGD^+^ and RGD^-^ Hemocytes

**Figure 5:**
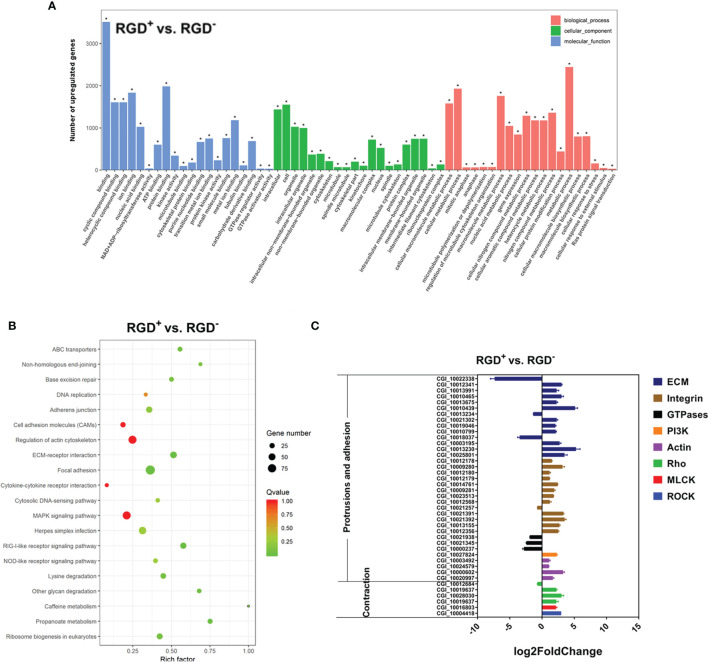
Transcriptome analysis of resting RGD^+^ hemocytes. **(A)** Top 60 GO terms enriched in the significantly upregulated genes in the resting RGD^+^ hemocytes compared to the resting RGD^-^ hemocytes. **(B)** Top 20 KEGG pathway items enriched in the significantly upregulated genes in the resting RGD^+^ hemocytes compared to the resting RGD^-^ hemocytes. **(C)** qPCR validation of the 40 selected DEGs involved in the formation of protrusions and firm adhesions at the front and contractions at the rear in resting RGD^+^ hemocytes compared to resting RGD^-^ hemocytes. RGD^+^ and RGD^-^ represent the resting RGD^+^ and RGD^-^ hemocytes, respectively. The asterisk (*) stands for statistical significance at *p* < 0.01.

A total of 13,292 DEGs were identified between the resting RGD^+^ hemocytes and RGD^-^ hemocytes, among which 6,767 genes were significantly more highly expressed and 6,525 genes were expressed at significantly lower levels in the resting RGD^+^ hemocytes than in the RGD^-^ hemocytes (**Figure S2A**). These DEGs were further annotated according to the GO and KEGG databases. A GO enrichment analysis of the top 60 significantly more highly expressed terms in the resting RGD^+^ hemocytes compared to those of the resting RGD^-^ hemocytes showed that 11 enriched GO terms were associated with cytoskeletal rearrangement and cell adhesion (**Figure 5A**). A KEGG pathway analysis revealed that 20 significantly higher-expressed items were also mainly related to cytoskeletal rearrangement and cell adhesion in the resting RGD^+^ hemocytes, including the “adherens junction,” “cell adhesion molecules (CAMs),” “regulation of actin cytoskeleton,” “ECM–receptor interaction,” and “focal adhesion” (**Figure 5B**).

Forty DEGs between the resting RGD^+^ and RGD^-^ hemocytes that have been reported to mediate cell migration were selected, and their expression levels were verified by qPCR (**Figure 5C**). Among them, three out of four types of genes that were reported to mediate protrusions and adhesion at the front edge of migrating cells were more highly expressed in resting RGD^+^ hemocytes than in resting RGD^-^ hemocytes, including ECM–integrin interaction-related genes, PI3Ks, actins, and GTPases. Specifically, 23 out of 27 ECM–integrin interaction-related genes, a PI3K gene, and four actin genes were expressed at significantly higher levels, while three small GTPase genes were expressed at lower levels in resting RGD^+^ hemocytes than in resting RGD^-^ hemocytes (**Figure 5C**). In addition, three types of genes, Rho, ROCK, and MLCK, reported to mediate contraction at the rear of migrating cells were also more highly expressed in resting RGD^+^ hemocytes than in resting RGD^-^ hemocytes, including two Rho genes, a ROCK gene and a MLCK gene (**Figure 5C**). These results showed that the genes reported to be involved in the protrusions and adhesion at the front edge and the contraction at the rear of migrating cells were overall more highly expressed in the resting RGD^+^ hemocytes than in the resting RGD^-^ hemocytes.

### 3.5 The Molecular Basis for the Enhanced Migration of RGD^+^ Hemocytes After *V. splendidus* Challenge

Given that the migration rate of RGD^+^ hemocytes but not RGD^-^ hemocytes significantly increased after *V. splendidus* challenge, transcriptome data from activated RGD^+^ hemocytes and resting RGD^+^ hemocytes were also comparatively analyzed to explore the molecular basis for the enhanced migration activity of RGD^+^ hemocytes after *V. splendidus* challenge. Unexpectedly, in addition to the conserved genes reported to be involved in cell migration, the neuroendocrine system genes also showed overall upregulated expression levels in RGD^+^ hemocytes after *V. splendidus* challenge. The results are summarized as follows.

#### 3.5.1 Conserved Genes Involved in Cell Migration

**Figure 6:**
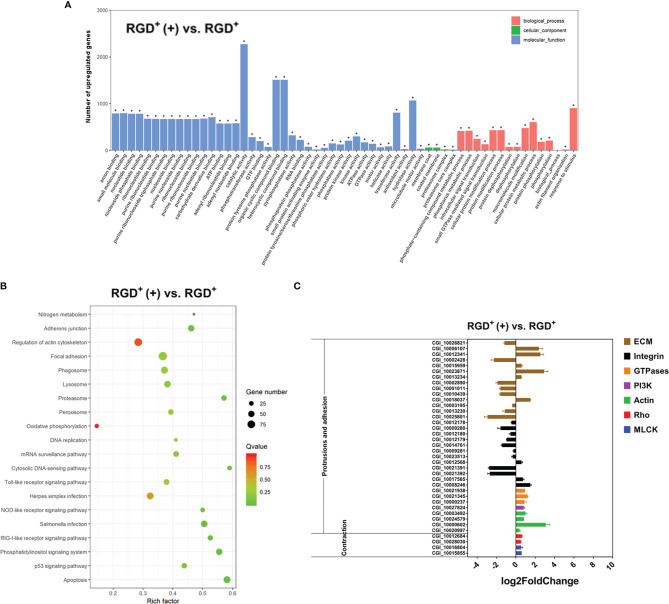
Transcriptome analysis revealing the molecular basis of the enhanced *C. gigas* RGD^+^ hemocyte migration in response to *V. splendidus* infection. **(A)** Top 60 GO terms enriched in the significantly upregulated genes in the activated RGD^+^ hemocytes compared to the resting RGD^+^ hemocytes. **(B)** Top 20 KEGG pathway items enriched in the significantly upregulated genes in the activated RGD^+^ hemocytes compared to the resting RGD^+^ hemocytes. **(C)** qPCR validation of the selected 38 DEGs involved in the formation of protrusions and firm adhesions at the front and contractions at the rear in the activated RGD^+^ hemocytes compared to the resting RGD^+^ hemocytes. RGD^+^ and RGD^+^ (+) represent the resting RGD^+^ and the activated RGD^+^ hemocytes, respectively. The asterisk (*) stands for statistical significance at *p* < 0.01.

Compared to those in the resting RGD^+^ hemocytes, 7,011 genes were significantly upregulated and 7,145 genes were significantly downregulated in activated RGD^+^ hemocytes (**Figure S2B**). GO analysis showed that three cell migration-related GO terms, namely, “small GTPase mediated signal transduction,” “GTPase activity,” and “motor activity,” were significantly enriched in the activated RGD^+^ hemocytes compared to those in the resting RGD^+^ hemocytes (**Figure 6A**). The KEGG pathway analysis also revealed that cell migration-related KEGG pathways, including adherens junction, regulation of actin cytoskeleton, and focal adhesion, were significantly enriched in the activated RGD^+^ hemocytes compared to those in the resting RGD^+^ hemocytes (**Figure 6B**).

Thirty-eight genes related to cell migration were selected from the transcriptome data from the activated RGD^+^ hemocytes and resting RGD^+^ hemocytes, and their expression levels were further verified by qPCR (**Figure 6C**). Among the genes reported to mediate the protrusions and adhesion at the front edge of migrating cells, four actin genes and a PI3K gene were upregulated in the activated RGD^+^ hemocytes compared to those in the resting RGD^+^ hemocytes (**Figure 6C**). Interestingly, three small GTPase genes were also all upregulated in the activated RGD^+^ hemocytes compared to those in the resting RGD^+^ hemocytes (**Figure 6C**). Most ECM–integrin interaction-related genes were downregulated in the activated RGD^+^ hemocytes compared to the resting RGD^+^ hemocytes, with the upregulation of 17 out of 26 genes. On the other hand, three types of molecules, namely, most Rho, ROCK, and MLCK genes associated with the contractions at the rear of migrating cells, were overall upregulated in the activated RGD^+^ hemocytes compared to those in the resting RGD^+^ hemocytes, including two Rho genes, a MLCK gene and a ROCK gene (**Figure 6C**).

#### 3.5.2 Neuroendocrine System Genes in Regulating Cell Migration

**Figure 7:**
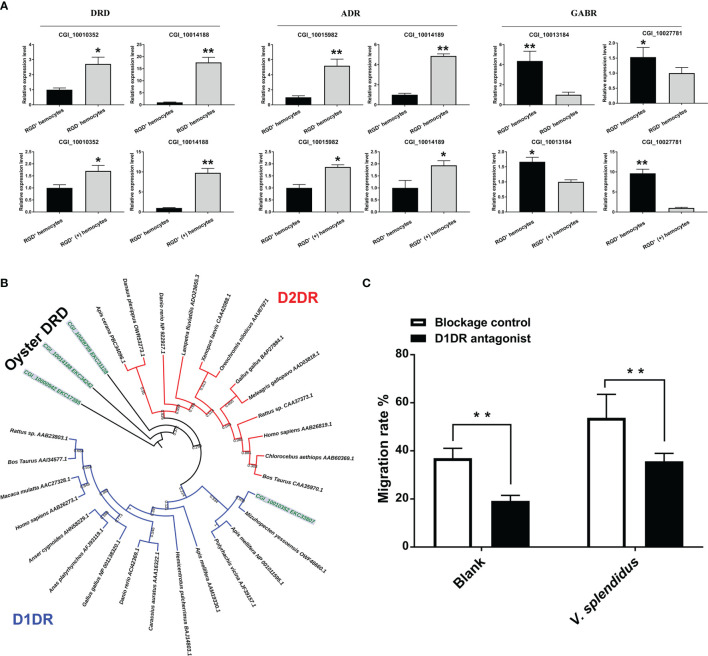
The involvement of the neuroendocrine system in regulating migration activity in response to *V. splendidus*. **(A)** qPCR validation of differentially expressed neuroendocrine factor receptor genes in resting RGD^+^ hemocytes compared to resting RGD^-^ hemocytes and in activated RGD^+^ (+) hemocytes compared to resting RGD^+^ hemocytes. **(B)** The dendrogram tree of the four differentially expressed DRDs. **(C)** Changes in migration activity after RGD^+^ hemocytes were blocked by a D1DR receptor inhibitor. The double asterisks (**) indicate statistical significance at *p* < 0.01, and the asterisk (*) indicates statistical significance at *p* < 0.05.

It has been reported in mammals that the neuroendocrine system plays a crucial role in regulating cell migration activity (59). The expression profiles of neuroendocrine system genes were analyzed to reveal the potential regulatory mechanism of the migration activity of RGD^+^ hemocytes after *V. splendidus* challenge. KEGG pathway analysis showed that the expression of excitatory neuroendocrine factor receptors, including four DRD (dopamine receptor D) genes and 12 ADR (adrenaline receptor) genes, was downregulated overall, while the expression of inhibitory neuroendocrine factor receptors, including three GABR (GABA receptor) genes, was upregulated in resting RGD^+^ hemocytes compared to that in the resting RGD^-^ hemocytes (**Figure S3**). In contrast, the expression of these excitatory neuroendocrine factor receptors was upregulated, while the expression of these inhibitory neuroendocrine factor receptors was downregulated overall in activated RGD^+^ hemocytes compared to that in the resting RGD^+^ hemocytes (**Figure S3**). Representative neuroendocrine factor receptor genes, including two DRD, two ADR, and two GABR genes, were selected, and their expression levels verified by qPCR were similar as those acquired through transcriptome data (**Figure 7A**). The expression of the excitatory neuroendocrine factor receptor genes in RGD^+^ hemocytes changed from an overall low level to a high level after the challenge with *V. splendidus*, while the expression of the inhibitory neuroendocrine factor receptor genes was the opposite, displaying a sophisticated neuroendocrine regulatory mechanism on the migration activity of RGD^+^ hemocytes.

To further verify the regulatory role of the neuroendocrine system in the migration activity of RGD^+^ hemocytes, a blocking assay of dopamine receptors was conducted. For the choice of effective agonists, the categories of four oyster dopamine receptors identified from transcriptome data were analyzed. According to the topological structure of the dendrogram, all dopamine receptors from various species could be clearly divided into two major categories, D1-type dopamine receptor (D1DR) and D2-type dopamine receptor (D2DR, **Figure 7B**). The four oyster dopamine receptors, in which CGI_10014188, CGI_10000942, and CGI_10028759 were grouped into a distinct branch, might be oyster-specific dopamine receptors, and CGI_10010352 could be well clustered with D1DR (**Figure 7B**). Therefore, the commercial agonist SCH 23390, a specific agonist of D1DR, was employed to block the RGD^+^ hemocytes. After agonist treatment, the migration rate of the resting RGD^+^ hemocytes significantly declined from 36.98% to 19.23% (*p* < 0.01, **Figure 7C**), and the migration rate of the activated RGD^+^ hemocytes also significantly decreased (53.75% vs. 35.56%, *p* < 0.01, **Figure 7C**). These results confirmed that excitatory neuroendocrine factors such as dopamine exerted an accelerative role in the migration of RGD^+^ hemocytes.

### 3.6 The Immunomodulatory Role of RGD^+^ Hemocytes in Antimicrobial Immunity

RGD^+^ hemocytes showed morphological features similar to those of agranulocytes and semigranulocytes in oysters (**Figure 3A**), which have been demonstrated to exert weak encapsulation and phagocytic activities and may not be the main immune killing hemocytes. Therefore, the expression profiles of immunomodulatory factors, including IL-17s, TNFs, and AMPs, were analyzed to reveal the immunomodulatory role of RGD^+^ hemocytes in the antimicrobial immunity of oysters. The results are summarized as follows.

#### 3.6.1 The Expression Profiles of Immunomodulatory Factor Genes in RGD^+^ Hemocytes

The expression levels of differentially expressed IL-17s and their receptor genes were overall downregulated in the resting RGD^+^ hemocytes with respect to the resting RGD^-^ hemocytes before *V. splendidus* challenge but significantly upregulated after *V. splendidus* challenge (**Figure S4**). Conversely, the expression levels of TNFs and their receptor genes were overall downregulated after *V. splendidus* challenge (**Figure S4**).

**Figure 8:**
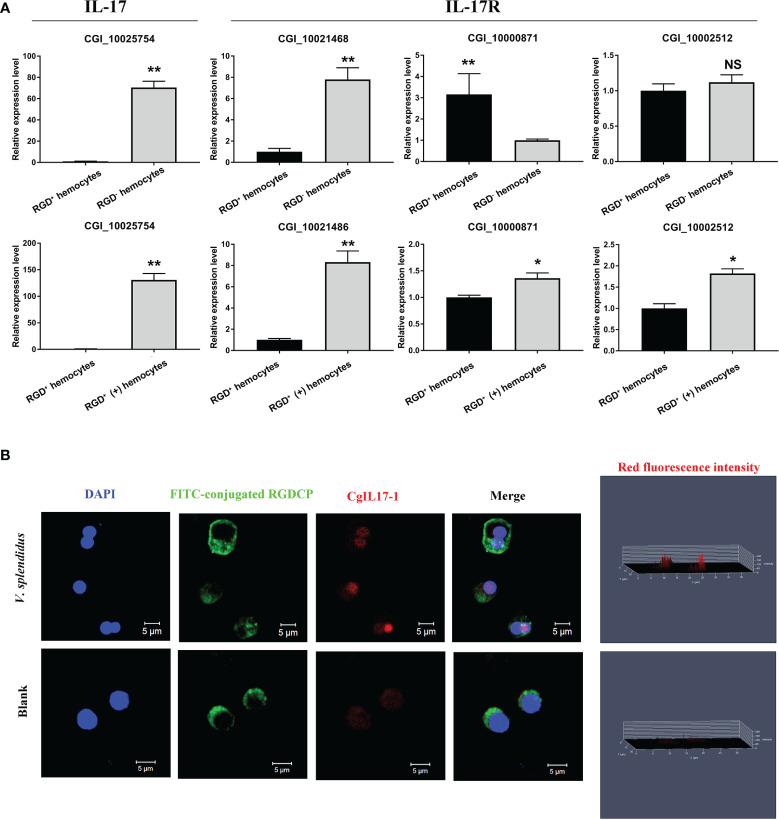
The expression profiles of IL-17s and their receptor genes. **(A)** qPCR validation of differentially expressed IL-17s and their receptor genes in resting RGD^+^ hemocytes compared to resting RGD^-^ hemocytes and in activated RGD^+^ hemocytes compared to resting RGD^+^ hemocytes. **(B)** Immunofluorescence validation of *Cg*IL17-1 (CGI_10025754) in activated RGD^+^ hemocytes compared to resting RGD^+^ hemocytes. FITC-conjugated RGDCP-labeled oyster integrins on cell membranes are indicated in green, the cell nucleus stained by DAPI is indicated in blue, and the *Cg*IL17-1 antibody conjugated with Alexa Fluor 594 is indicated in red. Bar = 5 μm. The asterisk (*) and the double asterisk (**) stand for statistical significance at *p* < 0.05 and *p* < 0.01 respectively and “NS” represents no statistical significance.

The representative IL-17 previously reported as *Cg*IL17-1 (CGI_10025754) (57) and three IL-17 receptor genes were selected, and their expression levels were verified by qPCR, with the results showing that the expression patterns of these genes were similar to those acquired through transcriptome data (**Figure 8A**). In addition, an immunofluorescence assay was performed to further detect the protein expression level of *Cg*IL17-1 in RGD^+^ hemocytes after *V. splendidus* challenge (**Figure 8B**). Although the protein content of *Cg*IL17-1 was low in oyster hemocytes, the analysis of fluorescent intensity showed that the protein level of *Cg*IL17-1 in the activated RGD^+^ hemocytes was significantly higher than that in the resting RGD^+^ hemocytes (**Figure 8B**). Consistent with the mRNA expression profiles, oyster IL17 proteins also showed a significant upregulation in RGD^+^ hemocytes after *V. splendidus* challenge, indicating that the IL-17 signaling pathway might play key immunomodulatory roles in RGD^+^ hemocytes.

#### 3.6.2 The Expression Profiles of AMP Genes in Oyster Hemocytes

**Figure 9:**
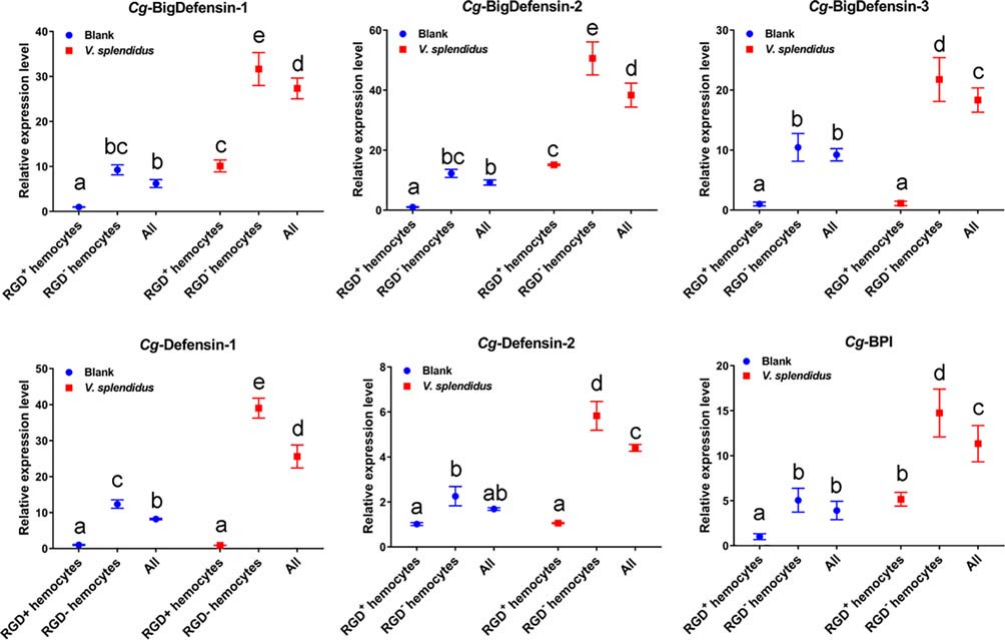
Changes in the mRNA expression of representative AMPs in different hemocyte populations before and after *V. splendidus* challenge. The relative expression levels of the reported AMPs, including *Cg*-BigDefensin-1, *Cg*-BigDefensin-2, *Cg*-BigDefensin-3, *Cg*-Defensin-1, *Cg*-Defensin-2, and *Cg*-BPI, in all hemocytes, RGD^+^ hemocytes, and RGD^-^ hemocytes were detected by qPCR. The letters “a, b, c, etc.” indicate statistical significance at *p* < 0.05.

IL-17 has been proven to promote the synthesis and expression of AMP genes, thereby enhancing host antimicrobial immunity (60). To investigate the roles of IL-17 in regulating antimicrobial immunity in RGD^+^ hemocytes, the expression profiles of six reported AMPs, *Cg*-Defensin-1~2, *Cg*-BigDefensin-1~3, and *Cg*-BPI, in different populations of oyster hemocytes were detected by qPCR (**Figure 9**). The results showed that these six AMPs all showed a significant upregulation (*p* < 0.05) in whole hemocytes after *V. splendidus* challenge (**Figure 9**). In detail, *Cg*-BPI, *Cg*-BigDefensin-1, and *Cg*-BigDefensin-2 showed a significantly increased (*p* < 0.05) expression in both RGD^+^ hemocytes and RGD^-^ hemocytes (**Figure 9**), and *Cg*-Defensin-1, *Cg*-Defensin-2, and *Cg*-BigDefensin-3 were also significantly (*p* < 0.05) upregulated in RGD^-^ hemocytes after *V. splendidus* challenge (**Figure 9**). Notably, the expression levels of all AMPs in RGD^+^ hemocytes were significantly lower (*p* < 0.05) than those in RGD^-^ hemocytes before or after *V. splendidus* challenge (**Figure 9**), which was consistent with the morphological observation that RGD^+^ hemocytes might not be the main immune killing hemocytes.

## 4 Discussion

In invertebrates, studies on hemocyte typing are greatly impeded due to the lack of appropriate cell lines and effective molecular markers (27). Although the migration of whole hemocytes has been observed after pathogen infections, it is still unclear which types or populations of hemocytes migrate at the infection sites to exert immune functions (23, 24). Here, FITC-conjugated RGDCP was used as the probe for RGD-binding integrins of *C. gigas*, which enabled a specific population of hemocytes, RGD^+^ hemocytes, to be marked with strong and clear fluorescent signals for cell sorting. Further assays were subsequently performed for the investigation of the cellular and molecular features of RGD^+^ hemocytes and revealed their potentially high migration activity and immunomodulatory roles against pathogen invasion. The strategy proposed here represents an important step forward in the functional study of specific hemocyte populations during pathogen infection in invertebrates.

The RGD^+^ hemocytes of *C. gigas* exhibited some distinct morphological features. First, Wright, Giemsa, and HE staining showed that RGD^+^ hemocytes were smaller, with a diameter of approximately 5–10 μm and with a lower C:N ratio and granularity. It seemed that RGD^+^ hemocytes shared similar morphological features with agranulocytes and semigranulocytes, which exerted weaker encapsulation and phagocytic activities than granulocytes and might not be the main immune killing hemocytes in *C. gigas* (61). Second, there were more MPO-positive signals in RGD^+^ hemocytes. MPO is a leukocyte-specific enzyme that catalyzes the formation of robust oxidants (62, 63). The interaction of integrins and their ligands, such as fibronectins, vitronectins, fibrinogens, and certain laminin isoforms that produce high levels of robust oxidants in cells, has been reported (64), so it is reasonable that the higher MPO level of RGD^+^ hemocytes is the result of integrin-mediated reactions in oyster hemocytes. Expectedly consistent with the higher MPO level in RGD^+^ hemocytes, it was confirmed that RGD^+^ hemocytes also had higher ROS levels in the subsequent detection. Third, the results also demonstrated that the level of Ca^2+^, an important messenger molecule (65), was higher in RGD^+^ hemocytes, suggesting frequent Ca^2+^-related cellular activities in RGD^+^ hemocytes. Notably, the percentages of RGD^+^ hemocytes significantly increased after the oysters were challenged with *V. splendidus*, suggesting the participation of RGD^+^ hemocytes in immune responses mainly mediated by integrins along with MPO and Ca^2+^.

Further transcriptome analysis revealed that the RGD^+^ hemocytes of *C. gigas* were equipped with a series of molecules mediating cell migration. It has been well accepted that the combined effect of protrusions and firm adhesions at the front and contractions and detachments at the rear is what makes the cell move forward (12). In the present study, the results revealed that a larger proportion of cell migration-related genes, including 23 out of 27 ECM–integrin interaction-related genes and PI3K and four actin genes, were highly expressed in RGD^+^ hemocytes before *V. splendidus* challenge. Correspondingly, ECM–integrin interaction-related molecules that are vital for the formation of tight adhesion points (66–68), PI3K, which is an activation molecule associated with migration-related signaling pathways (69), and actin, which is the central player in cell migration for rapid actin polymerization (70), have been proven to play different roles in the formation of protrusions and adhesions at the front of migrating cells (12). The results also showed that the tested two Rho, ROCK, and MLCK genes were highly expressed in RGD^+^ hemocytes before *V. splendidus* challenge. Rho, ROCK, and MLCK have been reported to be able to activate the molecular motor myosin to provide contractile forces, leading to contractions at the rear of migrating cells (71–73). Therefore, our transcriptomic results revealed that RGD^+^ hemocytes highly expressed a number of migration-related genes. In accordance with the transcriptomic results, the detection of migration rates of different populations of oyster hemocytes confirmed that the RGD^+^ hemocytes showed a higher migration activity before *V. splendidus* challenge, suggesting the potential migration capability of the RGD^+^ hemocytes even under resting conditions. To our knowledge, these findings represent the first molecular and functional evidence revealing a subpopulation of hemocytes with high migration activity in an invertebrate.

More importantly, the detection of migration rates showed that the migration activity enhanced by *V. splendidus* challenge only occurred in RGD^+^ hemocytes, indicating the specific functional characteristics of this hemocyte population. To investigate the molecular basis for the enhanced migration activity of RGD^+^ hemocytes after *V. splendidus* challenge, the transcriptome data from the activated RGD^+^ hemocytes and resting RGD^+^ hemocytes were further compared. Expectedly, the genes mediating the formation of protrusions and firm adhesions at the front of migrating cells, such as PI3K and actin (12), and the genes involved in the formation of contractions and detachments at the rear of migrating cells, such as Rho, ROCK, and MLCK (73), were upregulated in the activated RGD^+^ hemocytes compared to the resting RGD^+^ hemocytes. In addition, the expression levels of three tested small GTPases, including CDC42 and two Ras genes, were also upregulated in RGD^+^ hemocytes after *V. splendidus* challenge. Small GTPases such as Ras and CDC42 have been reported as core regulators of cell migration that associate with lipid membranes and act to choreograph molecular events and play critical roles in coordinating force generation by driving the formation of cellular protrusions as well as cell–cell and cell–matrix adhesions (74, 75). Therefore, the upregulation of small GTPase genes could be more favorable for RGD^+^ hemocyte migration after *V. splendidus* challenge. Moreover, 17 out of 26 DEGs associated with ECM–integrin interactions were downregulated in RGD^+^ hemocytes after *V. splendidus* challenge. Previous studies have shown in mammals that proper ECM–integrin interactions are vital for the formation of tight adhesion points, meaning that excessive ECM–integrin interactions increase the obstruction of mammalian leukocyte migration and inhibit cell migration (14, 76). Hence, the proper downregulation of ECM–integrin interaction genes was speculated to be more supporting for RGD^+^ hemocyte migration after *V. splendidus* challenge. Collectively, the upregulation of migration-promoting genes and downregulation of migration-inhibiting genes possibly together endowed RGD^+^ hemocytes with enhanced migration activity after *V. splendidus* infection.

Recently, an increasing number of studies in mammals have revealed that excitatory and inhibitory neuroendocrine factors can antagonistically regulate the activity of cell movement in addition to together supporting the behavior of animal movement (59, 77). Excitatory neuroendocrine factors such as L-adrenaline and L-dopamine induce the cell migration of embryonic neural progenitor cells through the activation of α1 adrenergic receptors and the dopamine D1 receptor, respectively (78). The role of inhibitory neuroendocrine factors in cell migration seems to be the opposite, with GABA inhibiting tumor cell migration dependent on Ca^2+^ (79). In *C. gigas*, circulating hemocytes can self-synthesize and secrete neuroendocrine factors, including dopamine, adrenaline, and GABA, which regulate multiple cellular immune processes, including cell phagocytosis and apoptosis (80–82). In the present study, transcriptomic analyses revealed that the expression of excitatory neuroendocrine factor receptor genes in RGD^+^ hemocytes changed from an overall low level to a high level after challenge with *V. splendidus*, while the expression change in inhibitory neuroendocrine factor receptor genes was the opposite. Correspondingly, a further blocking assay showed a significant decrease in the migration rate of RGD^+^ hemocytes after blocking the dopamine D1 receptor, demonstrating the stimulatory role of oyster dopamine signaling in cell migration like that in mammals. The regulatory role of other neuroendocrine factors, especially the inhibitory neuroendocrine factor GABA, in RGD^+^ hemocyte migration was not confirmed in this study, while the highly conserved roles of GABA and its synthetase in the neuroendocrine system have been revealed in oysters (83, 84). According to the observations on the roles of the mammalian neuroendocrine system in cell migration, the upregulation of excitatory neuroendocrine factor receptor genes and the downregulation of inhibitory neuroendocrine factor receptor genes in RGD^+^ hemocytes might also be linked to the enhanced migration activity of RGD^+^ hemocytes after *V. splendidus* challenge.

In mammals, leukocytes migrate to the infection sites to directly clear invading microbes and often produce various cytokines (e.g., TNFs and ILs) that regulate immune defense responses (3, 5). In the present study, RGD^+^ hemocytes showed the similar morphological features as those of agranulocytes and semigranulocytes in oysters, which are generally considered as non-immune killing hemocytes. Hence, the immunomodulatory roles they played after migrating to the infection sites were further evaluated. First, the expression profiles of important immunomodulatory factors, including TNFs, IL-17s, and their receptor genes, from transcriptome data were systematically analyzed in RGD^+^ hemocytes before and after *V. splendidus* challenge. The results showed that the expression of IL-17s and their receptor genes was overall low in resting RGD^+^ hemocytes before *V. splendidus* challenge but significantly upregulated after *V. splendidus* challenge. Immunofluorescence analysis further demonstrated the upregulation of the CgIL-17-1 protein in RGD^+^ hemocytes after *V. splendidus* challenge. Conversely, the expression levels of TNFs and their receptor genes were overall downregulated after *V. splendidus* challenge. These results indicated the specific accumulation of IL-17s and their receptors in RGD^+^ hemocytes following *V. splendidus* challenge. IL-17 is initially characterized by a proinflammatory role in inflammatory responses induced by pathogen infections, while it has been recently reported to promote the synthesis and expression of AMPs, thereby enhancing antimicrobial immunity (60). For example, in mice, the upregulation and release of IL-17A and IL-17F in neutrophils can induce the expression of AMPs in epithelial cells to withstand *Candida albicans*, which represents the paracrine regulation pattern of IL-17 for AMPs (85). Archer et al. also confirmed that IL-17A and IL-17F were critical for AMP production and clearance of *Staphylococcus aureus* in mice (86). In *C. gigas*, the upregulated co-expression of four *Cg*IL-17s and *Cg*-BigDefesin-1 has been observed after LPS or *V. splendidus* stimulation (87, 88). The silencing of the transcription factor *Cg*Rel by RNAi can not only cause the downregulation of *Cg*IL-17 expression but also hamper the expression of AMPs (88). The above evidence supports the conserved roles of IL-17s in regulating AMP production in both vertebrates and invertebrates. Therefore, the expression profiles of all six reported AMPs were further analyzed to reveal the immune functions of RGD^+^ hemocytes highly expressing IL-17s. The results showed that the expression of all six tested AMPs was significantly upregulated in whole hemocytes, especially in RGD^-^ hemocytes, representing the majority of oyster hemocytes after *V. splendidus* challenge, which shared a positive correlation with the induced increase in IL-17 gene expression in RGD^+^ hemocytes. These findings collectively suggested that RGD^+^ hemocytes might not be the main immune killing cells but a group of immunomodulatory cells with high migration activity in oysters.

**Figure 10:**
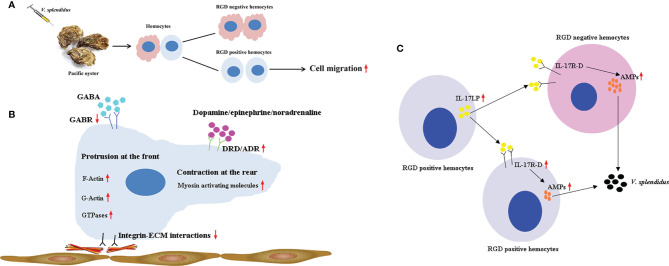
A supposed path to enhance antimicrobial immunity mediated by RGD^+^ hemocytes in *C. gigas*. **(A)** The migration activity of *C. gigas* RGD^+^ hemocytes was enhanced by stimulation with *V. splendidus*. **(B)** The molecular mechanisms for the mediation of high migration activity in *C. gigas* RGD^+^ hemocytes. The downregulation of integrin–ECM interactions and the upregulation of migration-promoting genes and migration-related genes enhanced the migration activity of RGD^+^ hemocytes in response to *V. splendidus* infection. In addition, the migration activity of RGD^+^ hemocytes was regulated by neuroendocrine factors, among which the excitatory neuroendocrine factor dopamine especially promoted the migration of RGD^+^ hemocytes. **(C)** The molecular mechanisms for the regulation of antimicrobial immunity of *C. gigas* RGD^+^ hemocytes. RGD^+^ hemocytes highly expressed the immunomodulatory factor IL-17s and their receptor genes, which might promote the production of AMPs in whole hemocytes to enhance antimicrobial immunity.

In summary, a population of hemocytes in the oyster *C. gigas*, RGD^+^ hemocytes, was obtained and identified as a small group of cells with similar cellular morphology as agranulocytes and semigranulocytes. RGD^+^ hemocytes highly expressed a series of genes related to cell migration to support their higher migration activity, and their migration activity was further enhanced after *V. splendidus* challenge, probably due to the downregulation of migration inhibiting-related genes and the upregulation of migration promoting-related genes. The migration activity of RGD^+^ hemocytes might be also controlled by the cooperation of dopamine and GABA neuroendocrine systems during *V. splendidus* infection, among which the excitatory neuroendocrine factor dopamine ultimately promoted the migration of RGD^+^ hemocytes. Meanwhile, the gene expression data suggested that RGD^+^ hemocytes might exert an immunomodulatory role in *C. gigas*, with highly expressed IL-17s to promote AMP production in whole hemocytes after *V. splendidus* infection. These findings may offer new insights into the main types of migrating hemocytes and their immune defense mechanisms against microbial infection in *C. gigas* and add to the theoretical basis of cellular immunity mediated by RGD^+^ hemocytes in invertebrates (**Figure 10**).

## Data Availability Statement

The datasets presented in this study can be found in online repositories. The names of the repository/repositories and accession number(s) can be found below: NCBI, accession ID: PRJNA826541.

## Ethics Statement

All experiments involving animals reported in this study were approved by the Ethics Committee of the Institute of Oceanology, Chinese Academy of Sciences.

## Author Contributions

ZL, LQ, and LS conceived and designed the experiments and wrote the manuscript. LQ revised the manuscript. ZL performed the experiments and analyzed the data. LQ and QL contributed reagents, materials, and analysis tools. ZL, LW, WW, and ZQL contributed to the discussion. All authors contributed to the article and approved the submitted version.

## Funding

This research was supported by the National Natural Science Foundation of China (No. 32072999) and the High Technology Project (863 Program, No. 2014AA093501) from the Chinese Ministry of Science and Technology.

## Conflict of Interest

The authors declare that the research was conducted in the absence of any commercial or financial relationships that could be construed as a potential conflict of interest.

## Publisher’s Note

All claims expressed in this article are solely those of the authors and do not necessarily represent those of their affiliated organizations, or those of the publisher, the editors and the reviewers. Any product that may be evaluated in this article, or claim that may be made by its manufacturer, is not guaranteed or endorsed by the publisher.
