# Supplementary figures and images for: RGD-Labeled Hemocytes With High Migration Activity Display a Potential Immunomodulatory Role in the Pacific Oyster Crassostrea gigas
Source: Front Immunol. 2022 Jul 5;13:914899. doi: 10.3389/fimmu.2022.914899 (PMC9294365; doi:10.3389/fimmu.2022.914899)

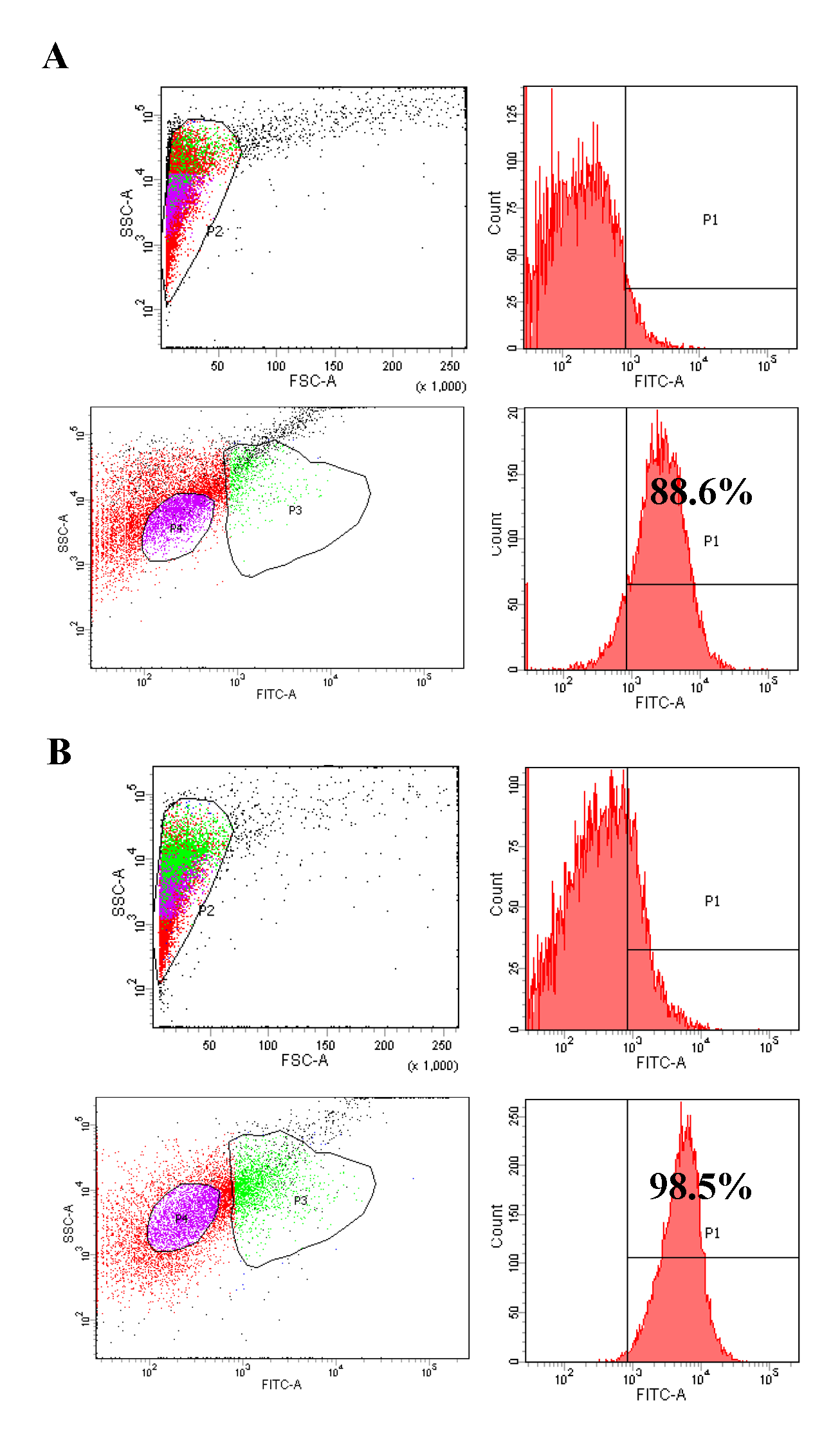

Supplement: Supplementary file 1 [file Image_1.tif]

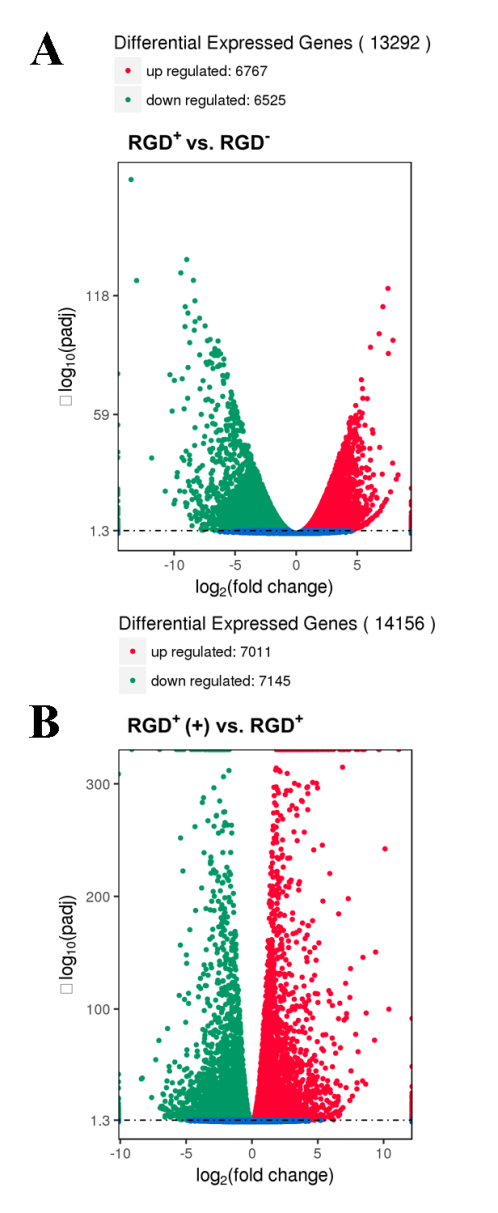

Supplement: Supplementary file 2 [file Image_2.tif]

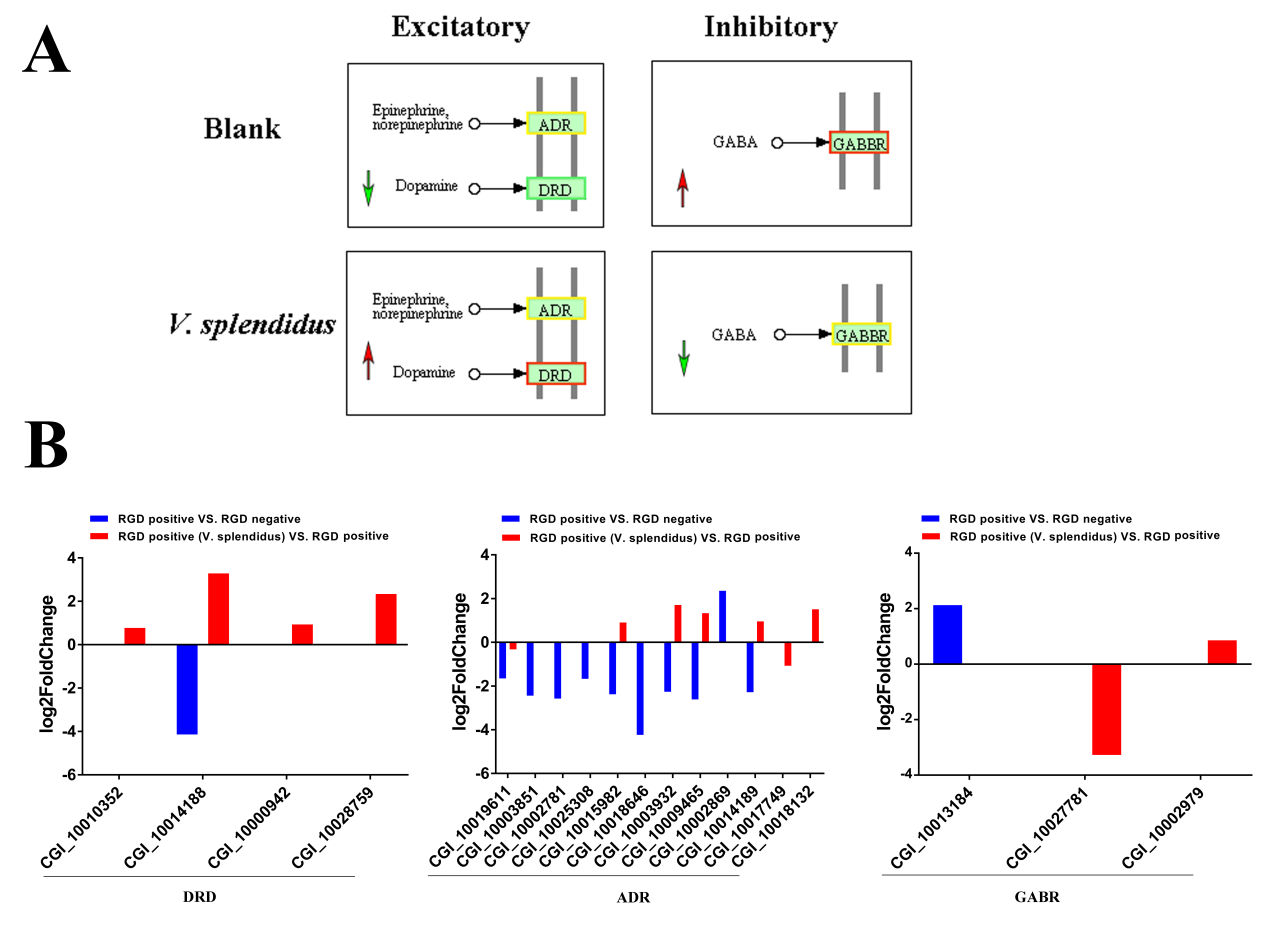

Supplement: Supplementary file 3 [file Image_3.tif]

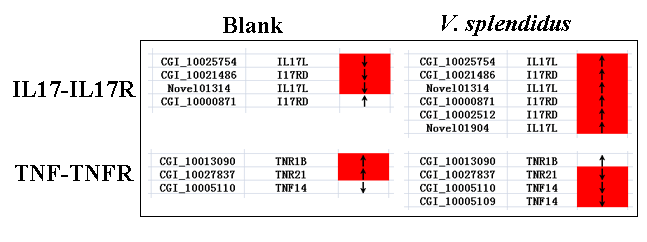

Supplement: Supplementary file 4 [file Image_4.tif]
