# Supplementary material for: RGD-Labeled Hemocytes With High Migration Activity Display a Potential Immunomodulatory Role in the Pacific Oyster Crassostrea gigas
Source: Front Immunol. 2022 Jul 5;13:914899. doi: 10.3389/fimmu.2022.914899 (PMC9294365; doi:10.3389/fimmu.2022.914899)
Supplement: Supplementary file 5 [file Table_1.docx]

**Table S1. Overall profiles of transcriptome sequencing.**

| **Sample name** | **Raw reads** | **Clean reads** | **Clean bases** | **Error rate (%)** | **Q20 (%)** | **Q30 (%)** | **GC content(%)** |
| --- | --- | --- | --- | --- | --- | --- | --- |
| **RGD^+^_1** | 48981606 | 47440128 | 7.12G | 0.02 | 98.72 | 95.79 | 39.3 |
| **RGD^+^_2** | 51740698 | 50692828 | 7.6G | 0.03 | 96.72 | 91.49 | 39 |
| **RGD^+^_3** | 56969438 | 55637472 | 8.35G | 0.03 | 96.86 | 91.68 | 37.06 |
| **RGD^+^(+)_1** | 53922048 | 52990832 | 7.95G | 0.03 | 96.76 | 91.66 | 41.25 |
| **RGD^+^(+)_2** | 57428746 | 56046684 | 8.41G | 0.03 | 96.63 | 91.4 | 41.25 |
| **RGD^+^(+)_3** | 49733092 | 48655340 | 7.3G | 0.03 | 96.43 | 91.02 | 41.23 |
| **RGD^-^_1** | 50683788 | 49723784 | 7.46G | 0.03 | 97.24 | 92.49 | 40.99 |
| **RGD^-^_2** | 54829856 | 53740516 | 8.06G | 0.03 | 97.29 | 92.64 | 41.36 |
| **RGD^-^_3** | 51354490 | 50506374 | 7.58G | 0.03 | 97.5 | 93.08 | 41.35 |

Note: RGD^+^ represents the resting RGD^+^ hemocytes; RGD^-^ represents the resting RGD^-^ hemocytes; RGD^+^(+) represents the activated RGD^+^ hemocytes.
