# Supplementary material for: RGD-Labeled Hemocytes With High Migration Activity Display a Potential Immunomodulatory Role in the Pacific Oyster Crassostrea gigas
Source: Front Immunol. 2022 Jul 5;13:914899. doi: 10.3389/fimmu.2022.914899 (PMC9294365; doi:10.3389/fimmu.2022.914899)
Supplement: Supplementary file 6 [file Table_2.docx]

**Table S2. Mapping of transcriptome sequencing reads.**

| **Sample_name** | **Total reads** | **Total**  **mapped** | **Multiple mapped** | **Uniquely**  **mapped** |
| --- | --- | --- | --- | --- |
| **RGD^+^_1** | 47440128 | 38377287  (80.9%) | 3173709 (6.69%) | 35203578  (74.21%) |
| **RGD^+^_2** | 50692828 | 40137002 (79.18%) | 3198800 (6.31%) | 36938202  (72.87%) |
| **RGD^+^_3** | 55637472 | 34043741 (61.19%) | 2254946 (4.05%) | 31788795  (57.14%) |
| **RGD^+^(+)_1** | 52990832 | 37715459 (71.17%) | 2470539 (4.66%) | 35244920  (66.51%) |
| **RGD^+^(+)_2** | 56046684 | 40371632 (72.03%) | 2669564 (4.76%) | 37702068  (67.27%) |
| **RGD^+^(+)_3** | 48655340 | 34636902 (71.19%) | 2275437 (4.68%) | 32361465  (66.51%) |
| **RGD^-^_1** | 49723784 | 38251337 (76.93%) | 2536607 (5.1%) | 35714730  (71.83%) |
| **RGD^-^_2** | 53740516 | 41508266 (77.24%) | 2824793 (5.26%) | 38683473  (71.98%) |
| **RGD^-^_3** | 50506374 | 39138132 (77.49%) | 2632295 (5.21%) | 36505837  (72.28%) |

Note: RGD^+^ represents the resting RGD^+^ hemocytes; RGD^-^ represents the resting RGD^-^ hemocytes; RGD^+^(+) represents the activated RGD^+^ hemocytes.
